# Gene expression patterns of chicken *neuregulin 3* in association with copy number variation and frameshift deletion

**DOI:** 10.1186/s12863-017-0537-z

**Published:** 2017-07-21

**Authors:** Hideaki Abe, Daiki Aoya, Hiro-aki Takeuchi, Miho Inoue-Murayama

**Affiliations:** 1 Wildlife Research Center, Kyoto University, 2-24 Tanaka-Sekiden-cho, Sakyo, Kyoto, 606-8203 Japan; 2 Akita Prefectural Livestock Experiment Station, 13-3 Kaisonumayachi, Jinguji, Daisen, Akita, 019–1701 Japan; 3 Department of Biological Science, Shizuoka University, 836 Ohya, Suruga, Shizuoka, 422–8529 Japan; 4 Wildlife Genome Collaborative Research Group, National Institute for Environmental Studies, 16-2 Onogawa, Tsukuba, Ibaraki, 305–8506 Japan

**Keywords:** Alternative splicing, Copy number variation (CNV), Frameshift, *Gallus gallus*, Indel, Isoform, Neuregulin 3 (NRG3), Premature stop codon, Retained intron, RT-qPCR

## Abstract

**Background:**

Neuregulin 3 (NRG3) plays a key role in central nervous system development and is a strong candidate for human mental disorders. Thus, genetic variation in *NRG3* may have some impact on a variety of phenotypes in non-mammalian vertebrates. Recently, genome-wide screening for short insertions and deletions in chicken (*Gallus gallus*) genomes has provided useful information about structural variation in functionally important genes. *NRG3* is one such gene that has a putative frameshift deletion in exon 2, resulting in premature termination of translation. Our aims were to characterize the structure of chicken *NRG3* and to compare expression patterns between *NRG3* isoforms.

**Results:**

Depending on the presence or absence of the 2-bp deletion in chicken *NRG3*, 3 breeds (red junglefowl [RJF], Boris Brown [BB], and Hinai-jidori [HJ]) were genotyped using flanking primers. In the commercial breeds (BB and HJ), approximately 45% of individuals had at least one exon 2 allele with the 2-bp deletion, whereas there was no deletion allele in RJF. The lack of a homozygous mutant indicated the existence of duplicated *NRG3* segments in the chicken genome. Indeed, highly conserved elements consisting of exon 1, intron 1, exon 2, and part of intron 2 were found in the reference RJF genome, and quantitative PCR detected copy number variation (CNV) between breeds as well as between individuals. The copy number of conserved elements was significantly higher in chicks harboring the 2-bp deletion in exon 2. We identified 7 novel transcript variants using total mRNA isolated from the amygdala. Novel isoforms were found to lack the exon 2 cassette, which probably harbored the premature termination codon. The relative transcription levels of the newly identified isoforms were almost the same between chick groups with and without the 2-bp deletion, while chicks with the deletion showed significant suppression of the expression of previously reported isoforms.

**Conclusions:**

A putative frameshift deletion and CNV in chicken *NRG3* are structural mutations that occurred before the establishment of commercial chicken lines. Our results further suggest that the putative frameshift deletion in exon 2 may potentially affect the expression level of particular isoforms of chicken *NRG3*.

**Electronic supplementary material:**

The online version of this article (doi:10.1186/s12863-017-0537-z) contains supplementary material, which is available to authorized users.

## Background

Recently, next generation sequencing has been used to identify insertion and deletion (indel) variation in chicken genomes [1]. The identification of short indels among 12 diverse chicken breeds has provided valuable information on genetic variation in genic, intergenic, and intronic regions. In terms of the identified coding indels, 1022 were predicted to cause frameshift mutations by non-triplet indels, leading to the generation of premature termination codons (PTCs). Indels, especially frameshift indels, are of great importance for their potential to alter gene function by the creation of alternative splicing events [2]. Our pilot survey showed that chicken *neuregulin 3* (*NRG3*) is one of these important genes that contain a putative frameshift mutation in a coding exon (Chr6; 3,200,391 in ENSGALG00000002327).

NRG3 is a neuronal-enriched growth factor that binds specifically to the ErbB4 receptor tyrosine kinase in the developing mammalian forebrain [3]. NRG3 plays multiple roles in the development of the embryotic central nervous system by regulating the migration and patterning of neural progenitor cells [4]. Similar to neuregulin 1 (NRG1), NRG3 has been identified as a strong candidate molecule for neurodevelopment disorders accompanied with cognitive and behavioral abnormalities [5–7].

Many studies have been carried out to determine the transcript variants of human *NRG3* cloned from various brain regions. *NRG3* undergoes extensive alternative splicing and utilization of its first and second exons [8, 9]. Two single nucleotide polymorphisms located in the 5′ region of *NRG3* were shown to have a critical effect on the selection of alternative first exons [10]. These findings on alternative exon selection, together with those derived from *NRG1* [11, 12], suggest that there may be alternative transcription start sites in the upstream region of chicken *NRG3*.

Our preliminary survey indicated the existence of a partial duplication (4.4 kb) of chicken *NRG3* in the reference genome assembly (galGal4), suggesting that copy number variation (CNV) may be another source of structural variation in this gene. CNV is the most prevalent type of structural variation that generally harbors relatively long duplications or deletions (≥1 kb) [13]. In the mouse genome, 5.5% of detected CNV overlapped with some part of a gene [14], thereby occasionally changing gene structure and transcription patterns. Accumulating evidence suggests that CNV loci harboring duplications or deletions have affected gene expression due to dosage compensation [15] or dosage sensitivity [16]. Even a partial gene duplication or deletion may have a pivotal impact on gene expression, especially when a CNV locus encompasses important sequence elements for transcription such as alternative transcription start sites and *cis*-regulatory factors [17].

Here, we present data from a comprehensive analysis of chicken *NRG3*, focusing on the association between structural variation and gene expression patterns. It is of great interest to examine whether a duplication that occurred in the ancestor of the chicken (i.e., red junglefowl [RJF]) would increase or decrease its copy number in commercial breeds under selective pressure, and whether increases and decreases in copy number would coincide with the up- or down-regulation of known *NRG3* isoforms.

## Results

### Structure of chicken *NRG3*

**Fig. 1:**
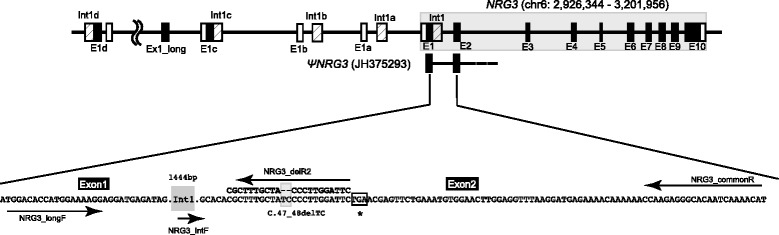
Schematic gene structure of chicken *NRG3.* Each coding exon and untranslated region is expressed as a black and open box, respectively. The shaded boxes indicate the 5′ conserved segments of intron 1 (330 bp in length). The size of each box and bar does not correspond to actual sequence length. Four blocks of duplicated exon 1 and flanking segments are shown in the upstream region of the gene (E1a/Int1a, E1b/Int1b, E1c/Int1c, and E1d/Int1d; see Table 1 for more details). Another duplicated segment found in an unknown chromosome of the Red Jungle Fowl genome (*ΨNRG3*; JH375293) is depicted under the schematic gene structure. The enlarged part indicates the positions where oligonucleotide primers were designed for genotyping and sequencing. A putative frameshift deletion (c. 47_48delTC) and premature stop codon are shown in light gray highlight and asterisk, respectively. The dark gray box (Int1) represents the entire intron 1

The structure of chicken *NRG3* (chr6: 2,926,344 − 3,201,956; ENSGALG00000002327) was investigated by BLAT search [18] against the RJF genome assembly (galGal4). The upstream sequence of chicken *NRG3* contained several highly conserved elements in the 5′ untranslated region (UTR), coding region of exon 1, and 5′ region of intron 1 (Fig. 1). Alignment of these elements with orthologous sequences showed preservation of the reading frame in the coding region (Additional file 1: Figure S1). Besides these elements, a 5′ truncated exon (“Exon1_long” embedded in the CLASS II isoforms) was found between E1c and E1d. The exact locations and sequence similarities of exon 1 and intron 1 are described in Table 1. In addition, a longer stretch of a duplicated element containing a highly conserved exon 1, intron 1, exon 2, and partial intron 2 (4.4 kb in total; hereafter, *ΨNRG3*) was found in an unknown location of the RJF reference genome (JH375293).

**Table 1:** Highly conserved elements found in the upstream region of chicken *NRG3*

	Region	Start	End	Length (bp)	Strand	Similarity (%)
*NRG3*	Exon 1	3,201,956	3,201,853	104	Reverse	
*NRG3*	Intron 1^a^	3,201,852	3,201,523	330	Reverse	
E1a	Exon 1	3,209,457	3,209,388	70^b^	Reverse	94.29
Int1a	Intron 1^a^	3,207,504	3,207,175	330	Reverse	94.85
E1b	Exon 1	3,217,198	3,217,129	70^b^	Reverse	97.14
Int1b	Intron 1^a^	3,215,224	3,214,895	330	Reverse	95.15
E1c	Exon 1	3,234,535	3,234,437	99	Reverse	95.56
Int1c	Intron 1^a^	3,234,436	3,234,120	317	Reverse	92.14
Ex1_long^c^	Exon 1	3,304,770	3,304,436	335	Reverse	99.70
E1d_1	Exon 1	5,485,492	5,485,523	32	Forward	93.75
E1d_2	Exon 1	5,485,525	5,485,551	27	Forward	96.30
E1d_3	Exon 1	5,485,879	5,485,912	34	Forward	97.06
Int1d	Intron 1^a^	5,485,913	5,486,242	330	Forward	95.15

^a^The exon 1 adjacent region (330 bp) is considered here, due to its high sequence similarity

^b^Only the 5′ UTR is highly conserved

^c^Truncated sequence of “Exon 1_long” in the CLASS II isoforms (see Fig. 4)

### Different frequencies of the 2-bp deletion allele among chicken breeds

**Fig. 2:**
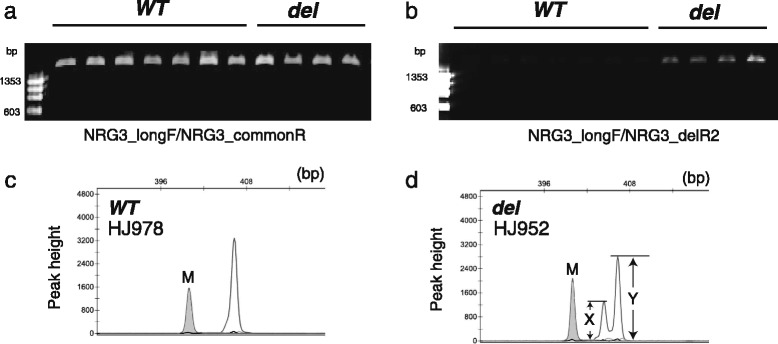
Genotyping of chicken *NRG3*. A different pattern of PCR amplification is shown in 1.5% agarose gel electrophoresis. **a** A set of primers was designed in exon 1 (NRG3_longF) and exon 2 (NRG3_commonR), and used for PCR. **b** A reverse primer (NRG3_delR2) overlapping with the putative frameshift deletion was used instead of NRG3_commonR. Note that much fainter bands can be seen in the chickens with the putative frameshift mutation (***del***) in panel b. Marker: ϕX174/*Hae*III digest. **c** Genotyping with a fluorescent primer generates a single dominant peak in wild-type chicken (***WT***). **d** A minor peak appears at a position 2-bp shorter than the dominant peak. The ratio of these peaks (X/Y) can be used to estimate the number of alleles with the putative frameshift deletion in a genome (see the Methods section for details). M: 400-bp size marker

Polymerase chain reaction (PCR) amplification using flanking primers (NRG3_longF and NRG3_commonR) generated a band with the expected size in all samples tested (Fig. 2a), while when an internal reverse primer (NRG3_dupR2) overlapping with the 2-bp deletion was used for amplification, faint bands appeared in several Boris Brown (BB) and Hinai-jidori (HJ) samples (Fig. 2b). PCR amplification with a fluorescently-labeled forward primer (NRG_intF) and Sanger sequencing confirmed that these faint bands had the 2-bp deletion in exon 2, as reported previously [1]. This PCR yielded 2 different patterns in peak detection: a single strong peak was detected in wild-type (hereafter, ***WT***; Fig. 2c), otherwise, a minor 2-bp shorter peak appeared with a much higher ***WT*** peak (hereafter, ***del***; Fig. 2d). The frequency of the ***del*** allele was largely different between commercial (BB and HJ) and primitive chickens (i.e., RJF). BB and HJ showed the same level of ***del*** frequency, whereas the ***del*** allele was not detected in RJF (Table 2).

**Table 2:** Chicken *NRG3* genotypes and copy number variation of partially duplicated segments

	Genotype				Copy Number	
Breed	*n*	male/female	*WT* (ratio)	*del*	Relative *ΨNRG3*	Estimated *del*
RJF	24	12/12	24 (1.00)	0 (0)	0.036	−
BB	44	44/0	24 (0.55)	20 (0.45)	0.111	4.030 (2.403^a^)
HJ	60	20/40	35 (0.58)	25 (0.42)	1.925	61.751 (5.250^a^)

*RJF* red junglefowl, *BB* Boris Brown, *HJ* Hinai-jidori

^a^The minimum value is assumed to have a single ***del*** allele

### Abundance of transcription factor binding sites in the conserved region of intron 1

LASAGNA-Search 2.0 [19, 20] identified a total of 1655 transcription factor binding sites (TFBSs) in intron 1 of chicken *NRG3*. The distribution of TFBSs was not biased in the highly conserved 5′ region of intron 1 using a 50-bp window (Additional file 2: Figure S2).

### CNV of chicken *NRG3*

**Fig. 3:**
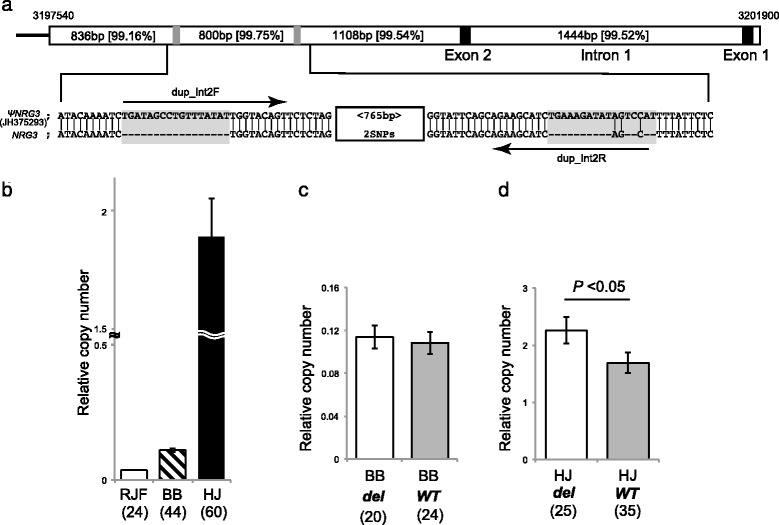
Sequence homology between chicken *NRG3* and duplicated segment in red junglefowl reference genome (RJF; JH375293). **a** Primers for quantitative PCR (qPCR) were designed in the regions where these 2 sequences are the least conserved (shown in gray highlights). The relative copy number of this duplicated segment was estimated by qPCR between chicken breeds as well as *NRG3* genotypes. Sequence similarities are shown in curly brackets with length. **b** Hinai-jidori (HJ) has a significantly higher number of copies than the other breeds (Dunn’s multiple comparisons test: *P* < 0.001). **c**, **d** Boris Brown (BB) and HJ display different patterns of copy number gains. In HJ, the relative copy number is different between the groups with the wild-type (***WT***) allele and with the putative frameshift deletion (***del***) in chicken *NRG3* (Mann–Whitney *U*-test: *P* < 0.05). The number in parentheses shows individuals from each group. Vertical bars represent the standard error of the mean

Quantitative PCR (qPCR) using a set of primers (dup_int2F and dup_int2R) specifically amplified the duplicated intron 2 in *ΨNRG3* (Fig. 3a). Relative copy number was largely different among the 3 chicken breeds: RJF showed the lowest copy number, while HJ had a significantly higher number of *ΨNRG3* than the other breeds (Dunn’s multiple comparisons test: RJF vs. HJ, *P* < 0.001; BB vs. HJ, *P* < 0.001; Fig. 3b). Assuming that all RJF chickens had a set of duplicated elements in the genome, the relative copy number of *ΨNRG3* was estimated to be 6.09 in BB and 34.75 in HJ. BB chicks showed no difference in *ΨNRG3* copy number between the ***del*** and ***WT*** groups (Fig. 3c), whereas the copy number of ***del*** alleles was significantly higher than that of ***WT*** alleles in HJ (Mann–Whitney *U*-test: *U* = 565.5, *z* = −1.91, *P* < 0.05; Fig. 3d). The number of ***del*** alleles was significantly different between BB and HJ (Mann–Whitney *U*-test: *U* = 97.5, *z* = 3.47, *P* < 0.001; Table 1; Additional file 3: Table S1).

### Structural variation of chicken *NRG3* transcripts

**Fig. 4:**
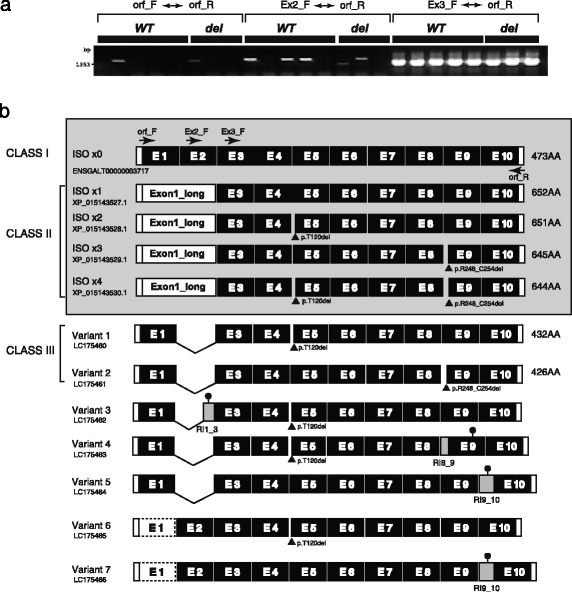
Schematic representation of transcript variation in chicken *NRG3*. **a** cDNA libraries derived from Hinai-jidori were amplified using 3 different sets of primers to estimate the approximate abundance of the 5′ end transcripts by agarose gel electrophoresis. **b** *NRG3* transcript variants are shown with the reference transcripts (highlighted). The first exon in each ISOx1 to ISOx4 transcript (Exon 1_long) is not annotated as a single independent exon in the galGal5 assembly (NC_006093.4; chr6: 3,826,268 − 3,826,962). The gray blocks depicted in variants 3, 4, 5, and 7 represent the retained introns of various lengths. Note that all transcripts with a retained intron generate a premature termination codon (PTC) in their downstream region. The PTCs and the presumed exon 1 in variants 7/8 are shown as filled circles and a dotted box, respectively

Complementary DNA (cDNA) libraries were constructed by reverse transcription using RNA specimens isolated from 5-day-old HJ chicks. PCR amplification of the cDNA libraries showed different gel electrophoresis patterns. The intensity of the PCR signal was obviously stronger when a forward primer was chosen in exon 3 than those designed in exons 1 and 2 (Fig. 4a). We isolated cDNA clones and determined the sequences of novel alternatively spliced transcripts in ***WT*** and ***del*** chicks. Variants 1 to 5 were identified from cDNA clones generated by orf_F/orf_R primers, while variants 6 and 7 originated from clones generated by the Ex2_F/orf_R primer set. Exon 2 skipping and intron retention were the major sources of the splice variation in chicken *NRG3* (Fig. 4b). Each isoform harboring the retained intron had a PTC that was produced by a shift in the reading frame. Variants 1 and 2 had no PTC in their mRNA sequences and were thereby classified as independent isoforms (class III). Nucleotide sequences for *NRG3* transcript variants were deposited in the GenBank database under the accession numbers LC175,460 − 1,755,466.

### Relative mRNA expression with or without the exon 2 cassette

**Fig. 5:**
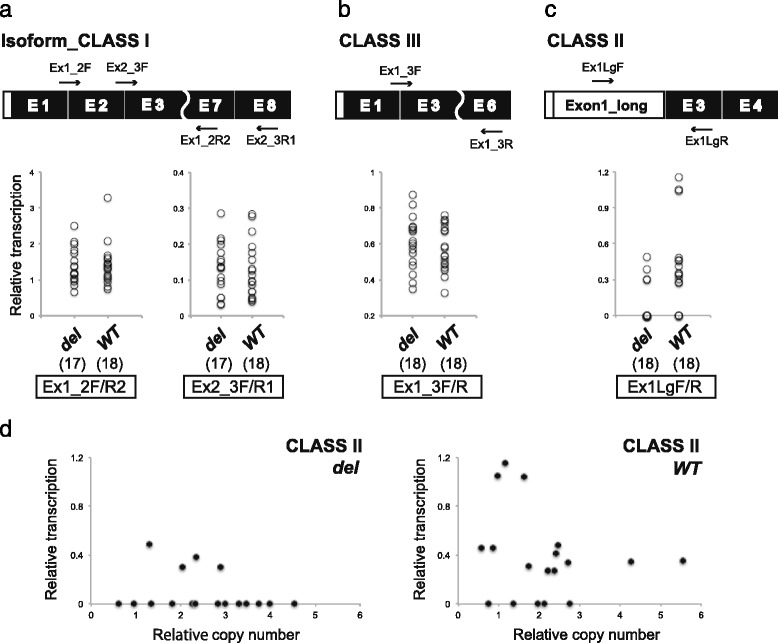
Comparisons of gene expression with or without the putative frameshift deletion in chicken *NRG3*. **a** Gene expression levels of the CLASS I isoform were compared between chicks with the wild-type (***WT***) allele and those with the deletion in exon 2 (***del***). **b**, **c** The same comparisons were made for the CLASS II and III isoforms. The number in parentheses shows individuals from each group. **d** Graphs indicate the relationship between relative mRNA expression of the CLASS II isoforms and relative copy number of *ΨNRG3*

Reverse transcription quantitative PCR (RT-qPCR) using exon-specific primers revealed different patterns of relative mRNA expression among the isoform classes. The CLASS I isoform showed no difference in relative expression between the ***del*** and ***WT*** groups (Fig. 5a). The CLASS III isoforms indicated a similar pattern of expression with CLASS I, even though the alternative exon 2 cassette was excluded (Fig. 5b). In the CLASS II isoforms, a significant difference in relative mRNA expression was detected between ***del*** and ***WT*** (Mann–Whitney *U*-test: *U* = 240, *z* = −2.45, *P* < 0.01; Fig. 5c). There was no correlation of relative gene expression for the CLASS II isoforms with the relative number of *ΨNRG3* between ***del*** and ***WT*** (***del***, *R* ^2^ = 0.055; ***WT***, *R* ^2^ = 0.042; Fig. 5d).

## Discussion

Chickens are inquisitive birds with intelligence surpassing that of many other domesticated animals. However, they are also sufficiently naive that panic can be induced by environmental noise, especially when they are kept in large numbers. The poultry industry has been searching for effective measures to prevent mass panic in cooperation with molecular scientists. We launched this research project to identify genes or genetic regions that are responsible for panic-like behavior in chicks; chicken *NRG3*, which has structural mutations in its coding region, is a strong candidate gene for this phenotype.

Yan et al. reported that a frameshift deletion in *NRG3* was observed in 5 chicken lines (White Leghorn, White Plymouth Rock, Tibetan, Beijing You, and Rhode Island Red) out of 12 breeds tested [1]. Although they used only 1 individual per breed for sequencing, these deletion alleles seem to be prevalent mainly in the commercial lines rather than in local breeds. The most plausible scenario for the evolution of chicken *NRG3* is that the first duplication of the 5′ part of *NRG3* (*ΨNRG3*) occurred in the ancestral population of RJF, followed by a rapid increase of its copy number in modern commercial lines. During this process, a frameshift mutation occurred in exon 2 and increased the number of alleles, coinciding with the rapid expansion of *ΨNRG3* in the chicken genome. Considering that Rhode Island Red has had a role in the creation of commercial laying strains, the *NRG3* frameshift deletion occurred before or during the line formation process. Intensive selection in a closed colony may help to maintain the frequency of deletion alleles and enable them to become prevalent in chicken breeds. Indeed, our previous study on array comparative hybridization demonstrated that commercial breeds of chicken have a considerable number of breed-specific CNV in their genomes [21]. Although many CNV loci are thought to be deleterious in the mammalian genome [22], the structural mutations identified in chicken *NRG3* seem to be favored by positive selection. To confirm these hypotheses, we need to increase the number of chicken breeds and specimens examined to trace evolutionary changes under selective pressure on the structure of *NRG3*.

In the present study we identified and characterized 2 structural mutations in chicken *NRG3*: a putative frameshift deletion in exon 2 (***del***) and CNV in the 5′ part of the gene. These structural mutations are not independent events, because the ***del*** alleles should be embedded in *ΨNRG3* according to our calculation. This finding is analogous with those obtained by studies of CNV in human *neutrophil cytosolic factor 1* (*NCF1*) and its pseudogenes [23]. Human *NCF1* has 2 duplicated copies with a 2-bp deletion in exon 2, which are transcribed differently in various human tissues. It is quite interesting to note that multiallelic CNV gives rise to most human variation in gene dosage and generates abundant variation in gene expression [24].

Alternative exon skipping events have been observed as potent sources of isoform variation in human *NRG3*. Several transcripts of chicken *NRG3* displayed skipping of exon 2, probably due to a frameshift with a PTC. However, no statistical difference was detected in relative gene expression between the ***WT*** and ***del*** groups for both CLASS I and III isoforms. If one assumes that the exon 2 skipping event corresponds to a frameshift deletion, CLASS III isoforms should be favored in the ***del*** group. A simple explanation for these observations would be the lack of the ***del*** allele in the true chicken *NRG3* gene. In this case, ***del*** alleles are scattered in only the recently duplicated copies in commercial lines (BB and HJ) and would not be involved in *NRG3* transcription. This is supported by the fact that exon 2 skipping occurred regardless of the absence of a frameshift deletion in ***WT*** chicks (Fig. 5b). Another important finding is that the ***del*** allele may suppress the expression of the CLASS II isoforms. The observation that the copy number of *ΨNRG3* is significantly larger in chickens with the ***del*** allele than in those with the ***WT*** allele supports the hypothesis that the biological relevance of the ***del*** allele is that it acts as a transcriptional regulator under positive selection.

It was somewhat surprising to find a highly conserved sequence block in the 5′ region of intron 1 upstream of chicken *NRG3* as well as in the duplicated segments (*ΨNRG3*). They show the same or higher levels of sequence conservation compared to the adjacent coding regions. Hence, introns, and especially the first introns, harbor evolutionarily constrained regulatory regions mediating both the level and complexity of gene expression [25]; the 5′ intron of chicken *NRG3* may contain transcriptional regulatory elements indispensable for splicing events. As our research did not show a biased distribution of TFBSs in the conserved region of intron 1, other regulatory factors such as histone modification might play an important role in cassette exon inclusion or skipping [26].

There are several studies suggesting that exon 2 of *NRG3* might affect the behavioral phenotypes of mice by changing its expression. Loo et al. generated *NRG3* mutant mice whose exon 2 was replaced with a neomycin cassette, and demonstrated that an increase in *NRG3* expression levels in the medial prefrontal cortex caused an increase in impulsive behavior [27]. Another study also targeting exon 2 of murine *NRG3* revealed that mutant mice display decreased freezing behavior and novelty-induced hyperactivity [28]. These findings are important because both hyperactivity and impulsive behaviors are the major tendencies observed in patients with schizophrenia and other mental disorders [29, 30]. Therefore, our findings on the naturally occurring mutations in chicken *NRG3* will pave the way for a better understanding of the relationship between *NRG3* structural variation coupled with altered expression in the brain and the abnormal behavior of animal models.

## Conclusions

This is the first study specifically examining the gene structure of *NRG3* in a non-mammalian vertebrate. The findings of the present study provide important information on the structural mutations that occur naturally in chicken *NRG3*. However, it is still puzzling how these structural components, including a putative frameshift deletion, CNV, alternative exons, and highly conserved introns, interplay with one another to orchestrate the complex expression pattern of *NRG3*. Regarding *NRG3* expression, our study has shown that exon 2 harboring a 2-bp deletion is associated with the downregulation of the expression of CLASS II isoforms. Gene expression profiling data collected from microarray or RNA sequencing will hold the key to elucidating further the genetic factors underlying gene expression heterogeneity in chicken *NRG3*.

## Methods

### Samples and DNA/RNA extraction

Three breeds of chicken were collected from different research facilities: Nagoya University (RJF; *n* = 24), Shizuoka University (BB; *n* = 44), and Akita Prefectural Livestock Experiment Station (HJ; *n* = 60). DNA was extracted either from blood (RJF/HJ) or liver tissue (BB) using commercial DNA extraction kits. Regarding HJ chicks, whole brain was removed from 5-day-old chicks to obtain RNA samples. The animals were decapitated rapidly just before sample collection. Each cerebrum was weighed and sliced into 1-mm-thick coronal sections at 4.0 mm from the postal edge of the cerebrum. Then, the amygdala regions were punched out with an 18 × ½ gauge blunt needle (NIPRO, Osaka, Japan) using the Atlas of the Chick Brain for reference [31]. Total RNA was extracted using an RNeasy® Mini Kit (QIAGEN, Tokyo, Japan). RNA concentration was measured with a Qubit™ RNA HS Assay Kit (Thermo Fisher Scientific, Tokyo, Japan), and adjusted to 50 ng/μL as templates for RT-PCR.

### Genotyping of the *NRG3* polymorphism

We designed primers that flanked the 2-bp deletion to perform conventional PCR (NRG3_longF and NRG3_commonR). Another reverse primer was designed in a position overlapping with the deletion in exon 2 (NRG3_delR2; Fig. 1). Primers were designed using Primer 3Plus online software (http://www.primer3plus.com) for the optimization of primer sequences. The sequences of all oligonucleotide primers used in this study are listed in Additional file 4: Table S2. In each case, PCR was carried out in a 15 μL reaction mixture containing G-Taq polymerase (Hokkaido System Science, Sapporo, Japan). The amplification conditions for PCR were: 95 °C for 2 min, then 30 cycles of 95 °C for 30 s, 60 °C for 30 s, and 72 °C for 1 min, with a final extension of 72 °C for 5 min. For genotyping, a forward primer (NRG3_IntF) was fluorescently labeled with 6-carboxyfluorescein (6-FAM), and used for PCR amplification under a slightly modified condition (72 °C for 30 s in the extension step). PCR products were analyzed on an ABI 3130xl Genetic Analyzer (Applied Biosystems, Tokyo, Japan) using the GeneScan® 500HD (ROX) size standard (Applied Biosystems).

### Search for TFBSs in conserved intron 1

We used LASAGNA-Search 2.0 [19, 20] to identify TFBSs in intron 1 of chicken *NRG3*. The JASPER core database was selected for searching matrices (cutoff *p*-value <0.01).

### qPCR for copy number estimation

To estimate the number of duplicated copies of *ΨNRG3* in the chicken genome, we designed a set of primers that bind specifically to the duplicate elements (dup_Int2F and dup_Int2R; see Fig. 2a). qPCR was performed using SYBR Premix ExTaq™ II (Takara, Ohtsu, Japan) and Thermal Cycler Dice Real Time System II (Takara). The amplification conditions for qPCR were: 95 °C for 30 s, then 40 cycles of 95 °C for 5 s, 60 °C for 30 s, and 72 °C for 30 s, followed by a melting curve step of 95 °C for 5 s, 60 °C for 30 s, and 95 °C for 15 s. After qPCR amplification, each PCR product was electrophoresed in a 2.0% agarose gel to confirm size and fidelity of product. In all cases, we obtained a single strong band of the expected size (Additional file 5: Figure S3). We used *β-actin* (*ACTB*) as a conventional reference gene for normalization. The relative copy number of *ΨNRG3* was calculated based on the standard curves generated by serial dilution of anonymous DNA as a template.

### Calculation of the number of *del* alleles in each chick

A combination of relative peak height in fluorescent genotyping and copy number estimated by qPCR was used to calculate the number of ***del*** alleles in each individual. Given that all RJF have a set of partially duplicated *NRG3* (*ΨNRG3*) in their genome, the number of ***del*** alleles can be calculated using the following equation:

$$ \mathrm{Number}\kern0.27em \mathrm{of}\kern0.27em \boldsymbol{del}\kern0.54em \mathrm{alleles}=\left(4\mathrm{X}\times {\mathrm{RCN}}_{\mathrm{samples}}\right)/\left(\mathrm{Y}\times \mathrm{RCN}\_\mathrm{RJF}\right), $$

where X is the peak height of the 2-bp shorter (***del***) allele, and Y is the peak height of the wild-type allele (see Fig. 2d). RCN stands for the relative copy number estimated by qPCR.

### Identification of isoform variation

To isolate transcript variations of chicken *NRG3*, RT-PCR was performed on each ***WT*** and ***del*** sample chosen from the HJ population. A cDNA library was constructed using 5 μg total RNA extracted from the amygdala. Reverse transcription was performed with a random oligo d(T)_18_ primer and the other reagents supplied in the PrimeScript™ RT-PCR Kit (Takara) according to the manufacturer’s instructions. Then, first strand cDNA was used for PCR amplification with oligonucleotide primers designed in the first and last exons (orfF and orfR; see Fig. 3b). PCR products were electrophoresed on a 1.5% agarose gel to check size and integrity. Newly synthesized cDNA was diluted with 18 μL distilled water and cloned into the pCR**®**2.1-TOPO**®** vector supplied in the TOPO**®** TA Cloning Kit (Life Technologies, Tokyo, Japan). *Escherichia coli* DH5α competent cells (Takara) were used for chemical transformation. Insertion was confirmed by PCR amplification with universal vector primers (M13) after plasmid preparation using a QIAprep® Spin Miniprep Kit (QIAGEN).

### Validation of relative gene expression

Two-step RT-qPCR was performed using the same SYBR amplification kits and real-time PCR machine described above. All forward oligonucleotide primers were designed in exon-exon junctions, except for “Exon1_long,” as shown in Fig. 4. We used the same set of *ACTB* primers with the above-mentioned qPCR assay for reference gene amplification. Relative mRNA expression was evaluated by the same manner described above.

## Additional files

Additional file 1 **: Figure S1.** Alignments of duplicated exons 1 and 2 found in the upstream region of chicken *NRG3* and *ΨNRG3*. (PDF 400 kb)

Additional file 2 **: Figure S2.** Distribution of transcription factor binding sites in intron 1 of chicken *NRG3*. (PDF 568 kb)

Additional file 3 **: Table S1.** Estimated number of ***del*** alleles in each individual of the chicken breeds. (PDF 46 kb)

Additional file 4 **: Table S2.** List of the oligonucleotide primers used in this study. (PDF 45 kb)

Additional file 5 **: Figure S3.** Example electrophoresis gel after qPCR using dup_Int2F and dup_Int2R. (PDF 382 kb)

## Abbreviations

BB: Boris Brown
cDNA: Complementary DNA
CNV: Copy number variation
HJ: Hinai-jidori
Indel: Insertion and deletion
NRG3: Neuregulin 3
PCR: Polymerase chain reaction
PTC: Premature termination codon
qPCR: Quantitative PCR
RJF: Red junglefowl
RT-qPCR: Reverse transcription qPCR
TFBS: Transcription factor binding site
UTR: Untranslated region
WT: Wild-type
