# Supplementary material for: Gene expression patterns of chicken neuregulin 3 in association with copy number variation and frameshift deletion
Source: BMC Genet. 2017 Jul 21;18:69. doi: 10.1186/s12863-017-0537-z (PMC5521077; doi:10.1186/s12863-017-0537-z)
Supplement: Supplementary file 1 — : Figure S1. Alignments of duplicated exons 1 and 2 found in the upstream region of chicken NRG3 and ΨNRG3. (PDF 400 kb) [file 12863_2017_537_MOESM1_ESM.pdf]

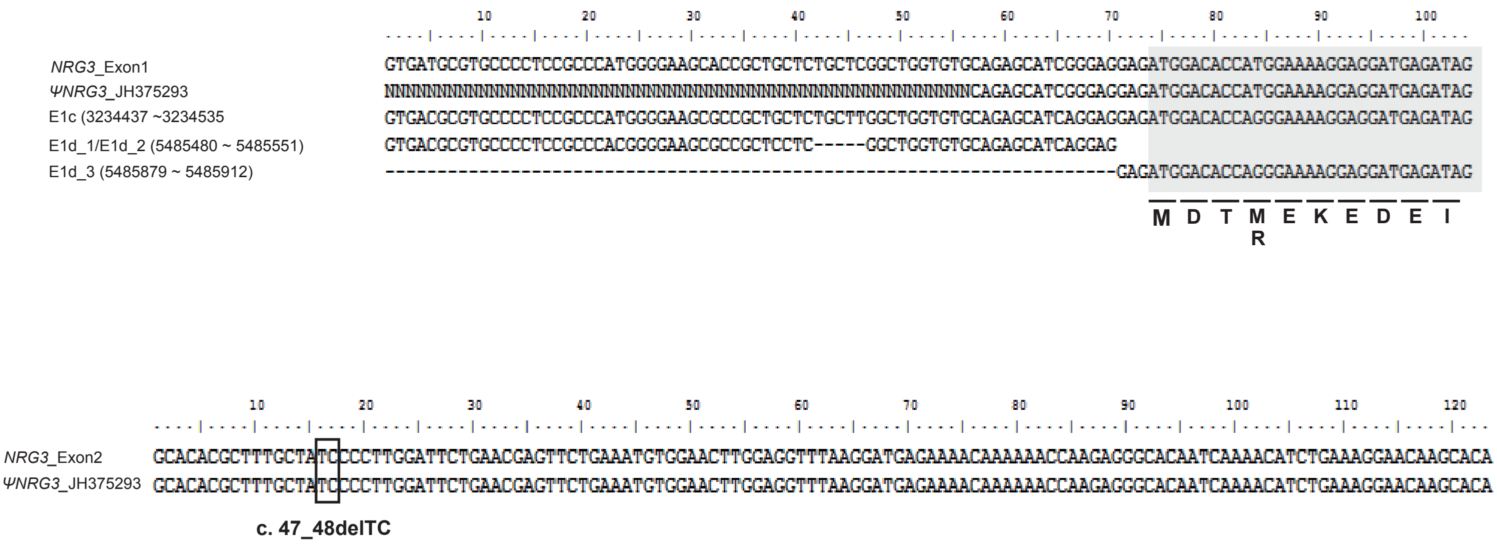
NRG3_Exon1
ΨNRG3_JH375293
E1c (3234437 ~3234535
E1d_1/E1d_2 (5485480 ~ 5485551)
E1d_3 (5485879 ~ 5485912)
M D T M E K E D E I
R
NRG3_Exon2
ΨNRG3_JH375293
c. 47_48delTC
