# Supplementary figures and images for: Gene expression patterns of chicken neuregulin 3 in association with copy number variation and frameshift deletion
Source: BMC Genet. 2017 Jul 21;18:69. doi: 10.1186/s12863-017-0537-z (PMC5521077; doi:10.1186/s12863-017-0537-z)

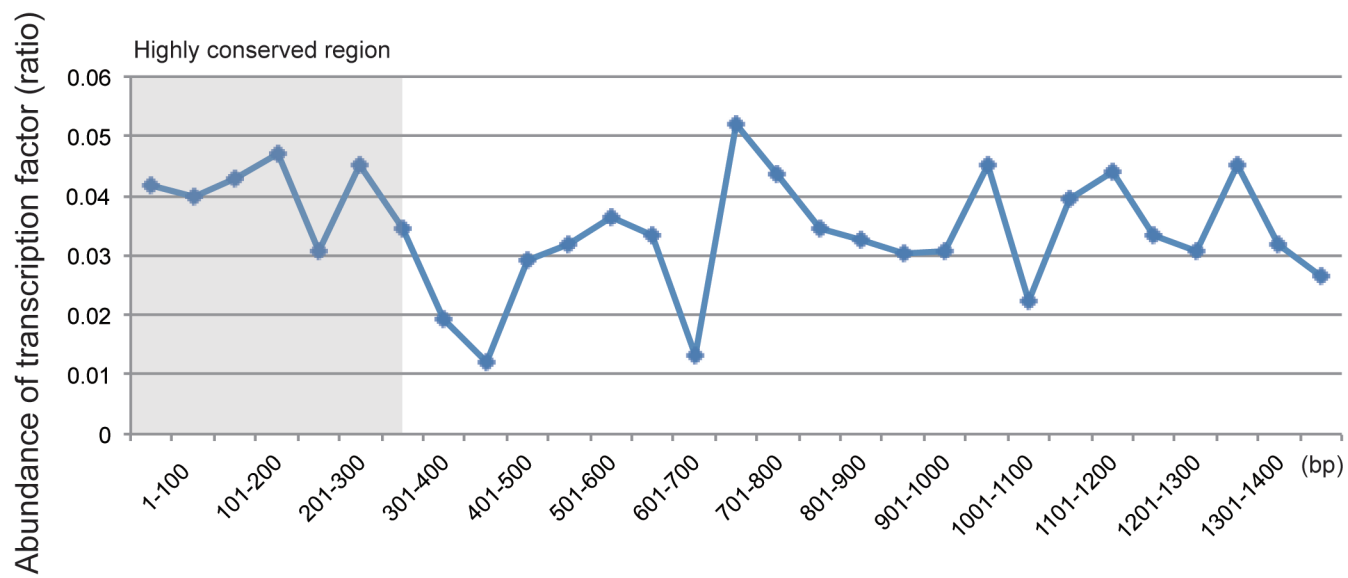

Supplement: Supplementary file 2 — : Figure S2. Distribution of transcription factor binding sites in intron 1 of chicken NRG3. (PDF 568 kb) [file 12863_2017_537_MOESM2_ESM.pdf]

770 bp→

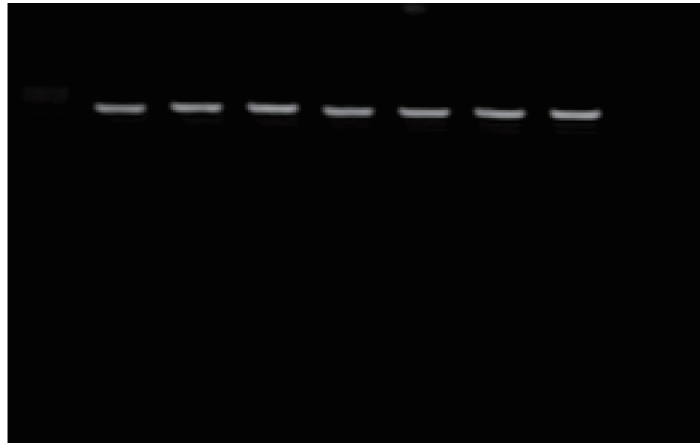

2% agarose gel

Supplement: Supplementary file 5 — : Figure S3. Example electrophoresis gel after qPCR using dup_Int2F and dup_Int2R. (PDF 382 kb) [file 12863_2017_537_MOESM5_ESM.pdf]
