# Supplementary material for: Gene expression patterns of chicken neuregulin 3 in association with copy number variation and frameshift deletion
Source: BMC Genet. 2017 Jul 21;18:69. doi: 10.1186/s12863-017-0537-z (PMC5521077; doi:10.1186/s12863-017-0537-z)
Supplement: Supplementary file 3 — : Table S1. Estimated number of del alleles in each individual of the chicken breeds. (PDF 46 kb) [file 12863_2017_537_MOESM3_ESM.pdf]

Table S1. Estimated Number of *del*+ Alleles in an Individual of Chicken Breeds

	Peak height		Relative copy number		***del*+**	***del*+**
ID	***del*+**	***WT***	sample	Average_RJF	copy number	mimimum = 1
BB387	588	2818	0.105980653	0.036387818	2.430898918	1.449339759
BB391	565	3006	0.121275993	0.036387818	2.505753139	1.49396901
BB393	828	1808	0.090444416	0.036387818	4.553209595	2.714694408
BB394	457	1300	0.100827642	0.036387818	3.896336262	2.323056306
BB396	555	1836	0.07450235	0.036387818	2.475678533	1.476038063
BB399	479	1441	0.216429564	0.036387818	7.908463042	4.71514872
BB404	822	2538	0.135668975	0.036387818	4.83019559	2.879837768
BB405	743	2027	0.077754406	0.036387818	3.133026335	1.867959051
BB407	644	1191	0.191708206	0.036387818	11.39511683	6.793945961
BB410	714	2476	0.222319984	0.036387818	7.047418254	4.20178042
BB412	811	1843	0.073342057	0.036387818	3.547746509	2.115221671
BB415	904	2676	0.127559685	0.036387818	4.736960179	2.824249365
BB416	1269	2854	0.080018226	0.036387818	3.911114656	2.331867414
BB417	354	1818	0.130000616	0.036387818	2.782651159	1.659059919
BB422	597	1939	0.09577963	0.036387818	3.241706175	1.932755662
BB424	385	1712	0.085869714	0.036387818	2.122760305	1.265622724
BB425	423	1484	0.070061447	0.036387818	2.19527808	1.30885895
BB426	878	3942	0.117225198	0.036387818	2.870138431	1.711221192
BB427	376	2490	0.101042533	0.036387818	1.677245727	1
BB428	1320	2544	0.058598344	0.036387818	3.342305478	1.992734532
HJ903	1125	3439	1.317712816	0.036387818	47.38545876	4.028693023
HJ908	293	1325	1.353607147	0.036387818	32.90397406	2.797482903
HJ849	439	1262	0.958819303	0.036387818	36.66451185	3.117202343
HJ985	682	3611	2.228820589	0.036387818	46.2738858	3.934187528
HJ938	1021	6483	2.884284959	0.036387818	49.93348284	4.245325026
HJ968	579	1655	4.54657732	0.036387818	174.8514024	14.8657973
HJ941	869	3052	3.752017359	0.036387818	117.4367596	9.984427004
HJ924	727	4581	2.265999703	0.036387818	39.53101276	3.360911121
HJ966	262	1106	3.465581678	0.036387818	90.24564983	7.672649571
HJ977	683	2361	1.827253114	0.036387818	58.10685906	4.94022225
HJ967	375	2006	2.315925504	0.036387818	47.5914457	4.046205951
HJ951	459	1895	3.988198415	0.036387818	106.1901419	9.028243999
HJ953	821	4002	3.003233765	0.036387818	67.726586	5.758087642
HJ983	650	3263	2.832505985	0.036387818	62.02561688	5.273393495
HJ934	623	2904	2.042115149	0.036387818	48.15879758	4.094441985
HJ973	1291	6564	0.60822916	0.036387818	13.1500947	1.118015867
HJ952	1321	2814	3.297656332	0.036387818	170.1719953	14.46795595
HJ928	994	6468	2.343444433	0.036387818	39.58904327	3.365844852
HJ940	520	3566	0.733761524	0.036387818	11.76199291	1
HJ749	468	1402	1.327907738	0.036387818	48.72700455	4.142750717
HJ784	1023	3125	1.039057802	0.036387818	37.39119051	3.178984277
HJ759	407	2030	0.841429719	0.036387818	18.54471619	1.57666446
HJ725	533	1966	0.967454855	0.036387818	28.83223952	2.451305638
HJ754	333	1738	2.388123482	0.036387818	50.29852082	4.276360411
HJ730	430	1922	4.077935446	0.036387818	100.2904066	8.526650831
