# Supplementary material for: Gene expression patterns of chicken neuregulin 3 in association with copy number variation and frameshift deletion
Source: BMC Genet. 2017 Jul 21;18:69. doi: 10.1186/s12863-017-0537-z (PMC5521077; doi:10.1186/s12863-017-0537-z)
Supplement: Supplementary file 4 — : Table S2. List of the oligonucleotide primers used in this study. (PDF 45 kb) [file 12863_2017_537_MOESM4_ESM.pdf]

Table S2. List of Oligonucleotide Primers Used in This Study

Name	5'-sequence-3'	Application	Label
NRG3_longF	TGGACACCATGGAAAAGGAGG	conventional PCR	–
NRG3_commonR	TGTTTTGATTGTGCCCTCTTGG	conventional PCR/genotyping	–
NRG3_shortF	AGCAGCCTGGAAGTAAGAACA	genotyping	5'-FAM
NRG3_del2R	GAATCCAAGGGTAGCAAAGCG	conventional PCR	–
orfF	CAGAGCATCGGGAGGAGATG	RT-PCR	–
orfR	TCACTTGGTCAATGCTGAGTCT	RT-PCR	–
Ex2fullF	ACACGCTTTGCTATCCCCTT	RT-PCR	–
Ex3F	CACTGAGCGATCTGAGCACT	RT-PCR	–
dup_Int2F	TGATAGCCTGTTTATATTGGTACAGT	qPCR	–
dup_Int2R	TGGACTATATCTTTCAGATGCTTCT	qPCR	–
ACTB_F	TGCGTGACATCAAGGAGAAG	qPCR /RT-qPCR (reference)	–
ACTB_R	CCAAGAAAGATGGCTGGAAG	qPCR /RT-qPCR (reference)	–
Ex1_2F	AGGATGAGATAGGCACACGC	RT-qPCR	–
Ex1_2_R2	GCCACACACTCTGACTTTGC	RT-qPCR	–
Ex2_3F	AGCACAATACTACAACGTATTCCAC	RT-qPCR	–
Ex2_3_R1	CAGGAGACAAAGCCTCTGGG	RT-qPCR	–
Ex1_3F3	TGAGATAGATACTACAACGTATTCCAC	RT-qPCR	–
Ex1_3R3	ACAAAGCATGCCCACTACGA	RT-qPCR	–
Ex1LgF	ACACGCTACCTCCTCCTACA	RT-qPCR	–
Ex1LgR	TCACAAAGCATTCCCCCTCA	RT-qPCR	–
